# Effect of sodium-glucose cotransporter-2 inhibitors on uric acid in patients with heart failure and preserved ejection fraction: a retrospective analysis in real word

**DOI:** 10.1186/s12872-026-05627-w

**Published:** 2026-02-14

**Authors:** Fangchao Lv, Dongming Zhang, Chenkai Xu, Xiaohong Xu

**Affiliations:** Department of Cardiology, Zhejiang Hospital, No. 12 Ling yin Road, Hangzhou, Zhejiang 310013 China

**Keywords:** Serum uric acid, Gout, HFpEF, SGLT-2 inhibitor

## Abstract

**Background and aims:**

While sodium-glucose cotransporter-2 inhibitors (SGLT-2i) are known to lower serum uric acid (SUA) in heart failure with reduced ejection fraction (HFrEF) or diabetes, their urate-lowering effect in heart failure with preserved ejection fraction (HFpEF) remains unclear. This study aimed to evaluate this effect in HFpEF patients.

**Methods:**

HFpEF patients newly treated with SGLT-2i were retrospectively included. Changes in SUA level and gout events were analyzed during follow-up.

**Results:**

We selected 734 patients according to propensity score matching, with the median age of 75.0(66.0–85.0) years. 31.9% were combined with chronic kidney disease(CKD) and the mean SUA was 6.4 ± 1.9 mg/dl. At 3 to 6-month follow-up, SGLT-2i treatment (79.6% dapagliflozin ) was associated with greater reduction of SUA (-0.91 ± 1.63 mg/dl vs. -0.10 ± 1.54 mg/dl; *p* < 0.001) and fasting glucose (-0.49(-1.59 to 0.37) vs. 0(-0.83 to 0.39) mmol/l, *p* = 0.002). 16 cases in SGLT-2i group had gout events and 23 cases in control group (*p* = 0.25). No significant difference was analyzed in estimated glomerular filtration rate (eGFR) change between groups. The control rate of SUA (< 6 mg/dl) was 72.2% and 51.2% respectively (*p* < 0.001). Multiple regression analysis suggested that changes of SUA were closely associated with gender, basal SUA level, eGFR and eGFR changes. Subgroup analysis showed that SGLT-2i could reduce SUA regardless of whether combined with diabetes or CKD, and the urate-lowering effect was more pronounced in those with underlying hyperuricemia.

**Conclusion:**

SGLT-2i (primarily dapagliflozin) could significantly reduce SUA level in HFpEF patients without increasing the risk of gout attack nor injuring renal function.

## Introduction

With the continuous development of social economy, heart failure (HF) has become a common and frequent-occurring disease. Heart failure with preserved ejection fraction (HFpEF) makes up about 50% of the overall HF population [1, 2], identified as left ventricular ejection fraction (LVEF) > 50%. Data from observational studies of community populations in western countries show that the in-hospital, 1-year and 5-year mortality of HFpEF patients were 2.4%-4.9%, 20%-29% and 53%-74%, respectively [2]. High prevalence rate and fatality rate make HFpEF a serious public health problem [3]. To identify and control the risk factors of HFpEF is an urgent need to prevent the deterioration of HF. The etiology of HFpEF is complex and is generally believed to be related with the increase of major risk factors like aging, obesity, diabetes mellitus (DM) and hypertension [1, 4].

Serum uric acid (SUA) is a final metabolite of purines, mainly excreted through the kidneys. Elevated SUA could cause hyperuricemia, which is considered as an important phenotype of HFpEF [5]. Hyperuricemia is thought to be closely related to the metabolic syndrome clustering of obesity, hypertension and DM, all of which are major risk factors for HFpEF [1, 4]. Hyperuricemia is also a common manifestation of HF. Japanese Cardiac Registry of Heart Failure report [6] shows that out-patients with SUA ≥ 7.0 mg/dL accounted for about 56%, suggesting that HF patients are more likely to complicate with hyperuricemia. Obesity, hypertension, diabetes, renal impairment and diuretic use are the main predisposition factors for hyperuricemia in those HF patients.

Besides, SUA is also a risk predictor of HFpEF, associated with the severity of HF and all-cause mortality [7]. A meta-analysis [8] including twenty-eight studies showed that for every 1 mg/dL increase in SUA, the odd of all-cause mortality risk increased by 4% and HF risk increased by 19%. Hyperuricemia can also cause gout attacks, which may increase the difficulty of clinical management of HF patients [9]. About 15%-20% of HF patients received urate-lowering therapy (ULT) prophylactically [10].

Recently, sodium-glucose cotransporter-2 inhibitors (SGLT-2i) have shown encouraging results in the treatment of HFpEF [11, 12], chronic kidney disease (CKD) [13] and DM [12]. As a novel oral hypoglycemic agent, SGLT-2i achieves hypoglycemic effects by inhibiting glucose reabsorption in the proximal renal tubules [14], and could improve the clinical outcomes of HF [11, 12], with or without DM [12].

SGLT-2i was also found to reduce SUA and the risk of gout attacks [15–18], which is an attractive versatile treatment option for patients with hyperuricemia. Studies mentioned above are mostly focused on patients with DM [17, 18] and HFrEF [15, 16]. There are few studies on the urate-lowering effect of SGLT-2i in HFpEF patients. However, Palazzuoli et al. [19] found that the incidence of hyperuricemia was much higher in HFpEF patients and increased SUA was considered as the only significant predictor of endpoint events, which was not found in HFrEF patients.

We hypothesized that SGLT-2i could reduce SUA levels and lower the risk of gout attacks in HFpEF patients. In this single-center, retrospective study, we aimed to analyze the real-world efficacy of SGLT-2i in reducing SUA and gout events in HFpEF patients, and to explore whether its efficacy is associated with baseline SUA, renal function, or diabetes status. Notably, by leveraging data from the China Heart Failure Database, our study provides real-world validation in an unselected, comorbid HFpEF cohort and delivers population-specific evidence on SUA management in Chinese patients, thereby supplementing the existing evidence base.

## Methods

### Study population

We conducted a retrospective analysis of HFpEF patients who were hospitalized from January 2021 to August 2024 at the Heart Failure Center of Zhejiang Hospital. These patients were part of the China Cardiovascular Association Database- Heart Failure Center Registry, a nationwide, multicenter study of HF in China [20].

Review of the electronic health records was performed to select eligible patients as follows: ① symptoms of HF, like chest tightness, shortness of breath and fatigue (New York Heart Association class II-IV); ② increased natriuretic peptides level〔BNP ≥ 35 pg/mL or NT-proBNP ≥ 125 pg/ml in sinus rhythm, or BNP ≥ 105 pg/ml or NT-proBNP ≥ 365 pg/mL in atrial fibrillation(AF)〕;③LVEF ≥ 50%. Exclusion criteria were as follows: Previous LVEF < 40%, lack of SUA data, ongoing use of ULT and pre-admission SGLT-2i use.

### Data collection

According to the HF center protocol [20], patients were followed up via telephone or clinic visit at the 1-week, 1-month, 3-month, 6-month and 1-year after discharge. We collected data including history of comorbidities, laboratory variables and echocardiographic data at discharge and during 3 to 6-month follow-up of HFpEF patients. Gout attacks were also recorded during follow-up. We also screened the complete medical history of drugs that may affect UA levels, such as diuretics, aspirin, losartan, statins and angiotensin receptor-neprilysin inhibitor (ARNI).

### Outcomes

The study’s primary outcomes were the change of SUA level and control rate after administration of SGLT-2i. The secondary outcomes were gout events and changes in renal function during follow-up.

### Statistical analysis

We performed propensity score matching (PSM) to balance baseline characteristics between groups. Covariates incorporated into the propensity score model included clinically relevant confounders: sex, age, body mass index (BMI) and history of hypertension and DM. Matching was conducted using a 1:1 nearest-neighbor algorithm with a caliper width of 0.2 standard deviations of the logit propensity score. Descriptive statistics were applied, with continuous data shown as mean ± SD or median (IQR), and categorical data as n (%). Intergroup comparisons for categorical variables were made using the Chi-square or Fisher’s exact test. Normally distributed continuous variables were compared using the student’s t-test, while the Mann-Whitney U test was used for non-normally distributed variables. Baseline variables with a *p* < 0.1 (AF, BNP, FPG, HbA1C, use of ACEI/ARB/ARNI, ARNI, loop diuretic, metformin and sulfonylureas) and baseline eGFR (the main determinant of SUA levels) were entered into multivariable analysis. Multiple regression analysis was operated to assess the contributing factors (gender, DM, baseline SUA, baseline eGFR, ΔeGFR, ΔFPG, Loop diuretic) related to the SUA change. Subgroup analysis was performed according to gender, DM, baseline hyperuricemia and baseline eGFR. *P* < 0.05 was considered to be of statistically significance with a two-sided test. We used the IBM SPSS version 26.0 (SPSS Inc., Chicago, IL, USA) to perform the statistical analyses described above.

## Results

### Study sample

**Fig. 1:**
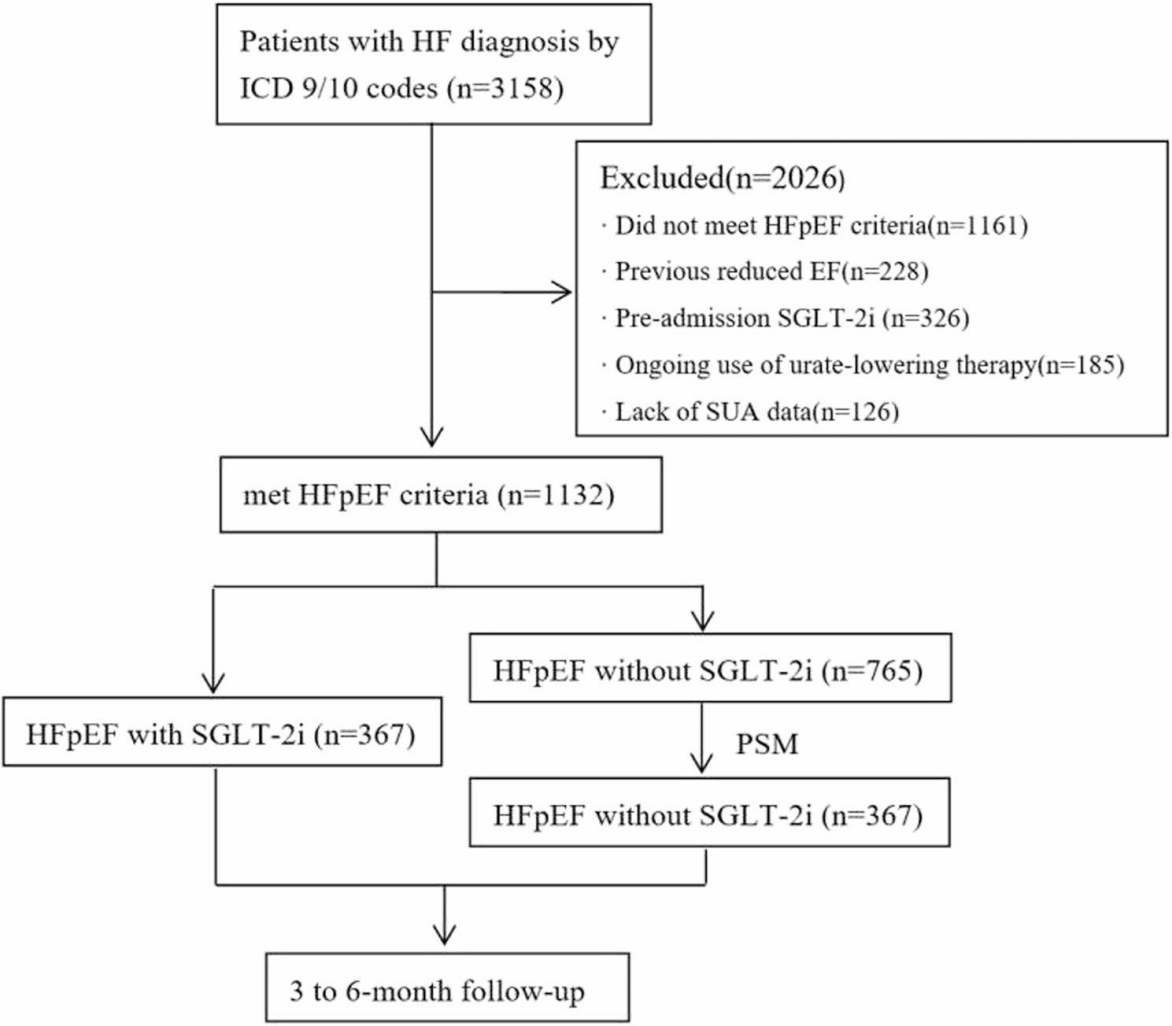
Flowchart depicting the participant selection process for the study. Abbreviations: HF, heart failure; ICD, international Classification of diseases; HFpEF, heart failure with preserved ejection fraction; EF, ejection fraction; SGLT-2i, sodium-glucose cotransporter-2 inhibitors; SUA, serum uric acid; PSM, propensity score matching

**Fig. 2:**
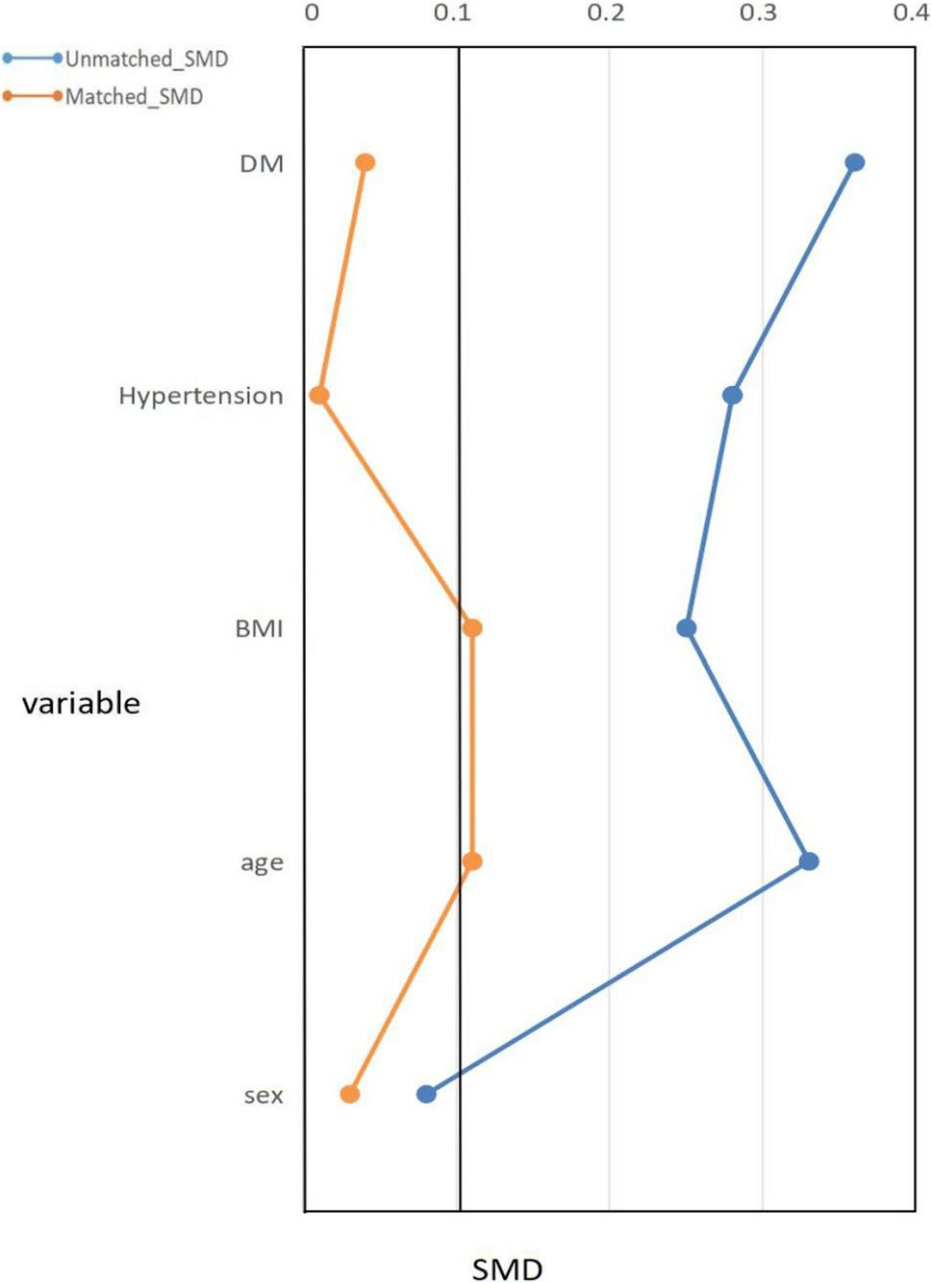
Propensity Score Matching. Abbreviations: BMI, body mass index; DM, diabetes mellitus; SMD, Standardized mean difference

A total of 3158 inpatients for acute HF from January 2021 to August 2024 in our department were identified from EHR. Flow diagram of participant selection process was showed in Fig. 1, among these HF patients, 1161 patients were excluded for not meeting the HFpEF inclusion criteria. Additional exclusions were as follows: 228 for a history of reduced EF, 326 for prior SGLT-2i use, 185 for ongoing ULT, and 126 for missing SUA data. Finally, 1132 patients were selected, including 367 newly added SGLT-2i patients. To balance the baseline data, we performed 1:1 PSM by sex, age, BMI and history of hypertension and DM (Fig. 2), then another 367 HFpEF patients without SGLT-2i use were selected as control group.

## Baseline characteristics

Table 1 showed the patients’ baseline characteristics. The mean age was 73.2 ± 14.4 years, with mean BMI 23.7 ± 3.6 kg/m^2^, and 62.0% were male. Patients enrolled were combined with more comorbidities, of whom 82.2% had two or more chronic diseases.

**Table 1:** Baseline characteristics for matched study population

	Total Population *n* = 734	SGLT-2i(+) *n* = 367	SGLT-2i(-) *n* = 367	*p* value before PSM	*p* value after PSM	SMD after PSM
Age, years	73.2 ± 14.4	72.9 ± 14.5	74.5 ± 13.7	0.075	0.13	0.11
Male sex, n (%)	455(62.0%)	225(61.3%)	230(62.7%)	0.90	0.70	0.03
Smoking	124(16.9%)	64(17.5%)	60(16.3%)	0.48	0.83	0.03
BMI, kg/m2	23.7 ± 3.6	24.0 ± 3.6	23.6 ± 3.5	0.042	0.37	0.11
Blood Pressure, mmHg						
Systolic	136.6 ± 23.0	135.5 ± 24.2	138.0 ± 22.2	0.28	0.49	0.11
Diastolic	74.1 ± 13.9	74.6 ± 13.6	73.8 ± 13.5	0.32	0.78	0.06
HR, bpm	76.8 ± 16.8	76.4 ± 17.2	77.2 ± 16.5	0.20	0.62	0.05
Comorbidities						
Hypertension	519(70.7%)	260(70.8%)	259(70.6%)	0.033	0.94	0.01
CAD	425(57.9%)	223(60.8%)	202(55.0%)	0.23	0.12	0.12
ACS	109(14.9%)	50(13.6%)	59(16.1%)	0.57	0.35	0.07
DM	250(34.1%)	122(33.2%)	128(34.9%)	0.012	0.64	0.04
Gout	39(5.3%)	22(6.0%)	17(4.6%)	0.86	0.41	0.06
AF	413(56.3%)	195(53.1%)	218(59.4%)	0.052	0.087	0.13
Stroke	81(11.0%)	43(11.7%)	38(10.4%)	0.78	0.56	0.04
CKD (stage III or higher)	231(31.5%)	110(30.0%)	121(33.0%)	0.094	0.34	0.07
Depression	33(4.5%)	17(4.6%)	22(6.0%)	0.65	0.41	0.06
COPD	79(10.8%)	39(10.6%)	48(13.1%)	0.91	0.31	0.08
Laboratory Data						
Hemoglobin, mg/dL	124.6 ± 21.0	125.7 ± 21.7	123.4 ± 20.2	0.46	0.14	0.11
Creatinine, mg/dL	98.4 ± 67.0	96.7 ± 56.3	100.1 + 76.3	0.53	0.49	0.05
eGFR, ml/min/1.73 m^2^	77.4 ± 34.2	74.4 ± 27.5	74.1 ± 30.1	0.078	0.80	0.01
BNP, pg/ml*	297.7 (180.0 to 471.6)	390.2 (188.9 to 655.3)	317.4 (191.2 to 489.4)	< 0.001	< 0.001	0.25
FPG, mmol/l	6.7 ± 2.9	6.9 ± 2.8	6.4 ± 2.5	0.010	0.006	0.19
HbA1c	6.6 ± 1.3	6.7 ± 1.4	6.4 ± 1.2	< 0.001	< 0.001	0.23
SUA, mg/dl	6.4 ± 1.9	6.4 ± 1.8	6.4 ± 1.9	0.65	0.56	0.00
LDL	2.0 ± 0.8	2.0 ± 0.8	1.9 ± 0.7	0.32	0.25	0.13
Echocardiographic Parameters						
LVEF, %*	58.0(55.1 to 63.1)	58.7(54.0 to 62.7)	58.7(55.8 to 63.9)	0.27	0.51	0.00
LVEDD, mm	47.9 ± 6.9	48.5 ± 7.9	47.6 ± 7.2	0.20	0.24	0.12
IVST, mm*	10.4(9.0 to 11.5)	10.1(8.8 to 11.4)	10.8(8.9 to 11.8)	0.22	0.65	0.34
Therapy						
Beta-Blocker	508(69.2%)	262(71.4%)	246(67.0%)	< 0.001	0.20	0.09
ACEI/ARB/ARNI	520(70.8%)	269(73.3%)	251(68.4%)	0.010	0.094	0.11
ARNI	397(54.1%)	211(57.5%)	186(50.7%)	0.021	0.064	0.14
SGLT-2i	367(50%)	367(100%)	-	NA	NA	NA
dapagliflozin	292(39.8%)	292(79.6%)	-	NA	NA	NA
Empagliflozin	67(9.1%)	67(18.2%)	-	NA	NA	NA
Others	8(1.1%)	8(2.2%)	-	NA	NA	NA
MRA	491(66.9%)	244(66.5%)	247(67.3%)	0.50	0.81	0.02
Loop Diuretic	516(70.3%)	269(73.3%)	247(67.3%)	< 0.001	0.076	0.02
Does of Loop Diuretic, mg/d	13.2 ± 10.4	13.6 ± 11.7	12.9 ± 9.1	NA	0.55	0.07
Antiplatelet drug	345(47.0%)	177(48.2%)	168(45.8%)	0.65	0.51	0.05
OACs	375(51.1%)	182(49.6%)	193(52.6%)	0.76	0.42	0.06
Statin	600(81.7%)	298(81.2%)	302(82.3%)	0.86	0.70	0.02
Other antidiabetic drug						
Metformin	124(16.9%)	67(18.3%)	89(15.5%)	< 0.001	0.058	0.07
DPP4i	99(13.5%)	48(13.1%)	51(13.9%)	0.051	0.75	0.02
Insulin	63(8.6%)	29(7.9%)	34(9.3%)	0.061	0.51	0.05
Sulfonylurea	65(8.9%)	24(6.5%)	41(11.2%)	0.013	0.027	0.16

*Abbreviations*: *SGLT-2i* sodium-glucose cotransporter-2 inhibitors, *SMD* Standardized Mean Difference, *BMI* body mass index, *HR* heart rate, *CAD* coronary artery disease, *ACS* acute coronary syndromes, *DM* diabetes mellitus, *AF* atrial fibrillation, *CKD* chronic kidney disease, *COPD* chronic obstructive pulmonary disease, *BNP* B-type natriuretic peptide, *FPG* Fasting Plasma Glucose, *SUA* serum uric acid, *LDL* low density lipoprotein cholesterol, *LVEF* left ventricular ejection fraction, *LVEDD* left ventricular end diastolic diameter, *IVS* Interventricular septal thickness, *ACEI/ARB/ARNI* angiotensin converting enzyme inhibitor/angiotensin receptor blocker/angiotensin receptor neprilysin inhibitor, *MRA* mineralocorticoid recept antagonist, *CCB* calcium channel blocker, *OACs* oral anticoagulants, *DPP4i* dipeptidyl peptidase 4 inhibitors

*median(IQR)

Laboratory findings at discharge revealed that 44.8% of the enrolled patients met the diagnostic criteria for hyperuricemia (defined as SUA ≥ 6.0 mg/dl in women and ≥ 7.0 mg/dl in men). Mean creatinine levels were 98.4 ± 67.0 mg/dl, and the average eGFR was 77.4 ± 34.2 mL/min/1.73 m². Additionally, 31.9% of the patients were classified as having chronic kidney disease (CKD) stage 3–5 (eGFR < 60 mL/min/1.73 m²). Following PSM, baseline characteristics were compared across groups. No statistically significant differences were observed in hemoglobin, SUA, creatinine, eGFR, or LVEF. However, significant differences were noted in BNP, fasting plasma glucose (FPG), and HbA1c, as summarized in Table 1.

Of the HFpEF patients newly treated with SGLT-2i in this cohort, dapagliflozin was used most frequently (79.6%), followed by empagliflozin (18.2%), as shown in Table 1. Other medication history was comparable between the groups for most drug classes, such as beta-blockers, diuretics, antiplatelet drugs, statins, and ARNI. The use of sulfonylureas, however, differed significantly.

### Outcomes

#### Change in SUA level

Table 2 details the changes in SUA and FPG at discharge and during follow-up. Reductions from baseline were observed for both parameters, and these decreases were more pronounced in the SGLT-2i group (primarily dapagliflozin), than in controls (SUA: -0.9±1.7 vs. -0.3±1.5 mg/dL, *p*<0.001; FPG: -0.8±2.1 vs.-0.4±2.0mmol/L, *p*=0.002). Correspondingly, the SGLT-2i group exhibited superior control rates for SUA targets of <6.0 mg/dL (72.2% vs. 51.2%) and <5.0 mg/dL (34.9% vs. 19.1%) (both *p*<0.001).

**Table 2:** Change in SUA levels from baseline to Post-Treatment

	SGLT-2i (+)				SGLT-2i (-)				inter-group *p* value	T/ Z value
	Baseline	Follow-up	Change	*p* value	Baseline	Follow-up	Change	*p* value		
SUA, mg/dl	6.4 ± 1.8	5.5 ± 1.3	-0.9 ± 1.7	< 0.001	6.4 ± 1.9	6.3 ± 1.5	-0.3 ± 1.5	0.001	< 0.001	5.17
FPG, mmol/l	6.9 ± 2.8	6.1 ± 1.6	-0.8 ± 2.1	< 0.001	6.4 ± 2.5	6.0 ± 1.9	-0.4 ± 2.0	< 0.001	0.002	3.06
eGFR, ml/min/1.73 m^2^	74.4 ± 27.5	71.4 ± 24.5	-2.4 ± 17.4	0.009	74.1 ± 30.1	73.3 ± 29.2	-1.4 ± 19.1	0.15	0.49	0.70
Control rate										
SUA < 6.0 mg/dl	265(72.2%)				188(51.2%)				< 0.001	5.98
SUA < 5.0 mg/dl	128(34.9%)				70(19.1%)				< 0.001	4.90
Gout event	16(4.4%)				23(6.3%)				0.25	1.15

*Abbreviations*: *SUA* serum uric acid, *SGLT-2i* Sodium glucose cotransporter-2 inhibition, *FPG* Fasting Plasma Glucose, *eGFR* estimated glomerular filtration rate

Multivariate analysis (shown in Table 3) showed that after adjusting for AF, BNP, FPG, HbA1C, GFR, use of ACEI/ARB/ARNI, loop diuretic , metformin and sulfonylureas, compared with control group, SUA decreased significantly after SGLT-2i administration (t=5.61, 95% CI 0.44-0.90; *p* <0.001, ηp²=0.042), which largely driven by dapagliflozin use.

**Table 3:** Multivariable analysis. (Adjusted for AF, BNP, FPG, HbA1C, eGFR, use of ACEI/ARB/ARNI, loop diuretic, Metformin and sulfonylureas)

	SGLT-2i (+) (*n* = 367)	SGLT-2i (-) (*n* = 367)	t	Adjusted difference between groups (95% CI)	*P* value	ηp²
ΔSUA, mg/dl	-0.9 ± 1.7	-0.3 ± 1.5	5.61	0.44 to 0.90	< 0.001	0.042
ΔFPG, mmol/l	-0.8 ± 2.1	-0.4 ± 2.0	1.15	-0.088 to 0.34	0.25	0.002
ΔeGFR, ml/min/1.73 m^2^	-2.4 ± 17.4	-1.4 ± 19.1	-0.053	-2.43 to 2.30	0.96	< 0.001
Gout event	16(4.4%)	23(6.3%)	0.83	-0.019 to 0.048	0.41	0.001

*Abbreviations*: *AF* atrial fibrillation, B-type natriuretic peptide, *FPG* Fasting Plasma Glucose, *eGFR* estimated glomerular filtration rate, *ACEI/ARB/ARNI* angiotensin converting enzyme inhibitor/angiotensin receptor blocker/angiotensin receptor neprilysin inhibitor, *SUA* serum uric acid, *ηp²* Partial Eta Squared

As shown in Table 4, multiple regression analysis revealed the association between male, DM, baseline SUA, eGFR, change of eGFR, change of FPG, dose of loop diuretic and change in SUA. The results showed that gender, baseline SUA, eGFR and change of eGFR had statistically significant effects on SUA change.

**Table 4:** Indicators of change in SUA level

	Univariate analysis			Multivariate analysis		
	β	t	*p* value	β	t	*p* value
Male	-0.002	-0.043	0.965	0.088	2.993	0.003
DM	0.025	0.678	0.498	0.016	0.580	0.562
Baseline SUA	-0.671	-24.459	< 0.001	-0.698	-23.505	< 0.001
eGFR	0.239	6.652	< 0.001	-0.159	-4.234	< 0.001
ΔeGFR	-0.252	-7.058	< 0.001	-0.189	-5.944	< 0.001
ΔFPG	0.035	0.936	0.349	0.035	1.288	0.198
Loop diuretic	-0.097	-2.644	0.008	-0.008	-0.271	0.787

*Abbreviations*: *DM* diabetes mellitus, *SUA* serum uric acid, *eGFR* estimated glomerular filtration rate, *FPG* Fasting Plasma Glucose

#### Gout event

Over the 3- to 6-month follow-up, the incidence of gout was comparable between the SGLT-2i group (*n* = 16) and the control group (*n* = 23), with no significant association found in either univariate (*p* = 0.25) or multivariate models (*p* = 0.26), as detailed in Tables 2 and 3. Furthermore, although a significant intra-group reduction in eGFR was noted in the SGLT-2i group (71.4 ± 24.5 vs. 74.4 ± 27.5 ml/min/1.73 m²; *p* = 0.009), the between-group comparison revealed no statistically significant difference in eGFR change.

### Subgroup analysis

We performed subgroup analyses within the SGLT-2i group according to gender, presence of DM, CKD, and baseline hyperuricemia. As presented in Table 5, SUA levels were lowered by SGLT-2i treatment irrespective of gender, DM, or CKD status. Notably, the urate-lowering effect was most robust among individuals with hyperuricemia at baseline.

**Table 5:** Subgroup analysis of SUA changes in patients with SGLT-2i

Subgroup	Patients with SGLT-2i						
	Baseline	Follow-up	Change	T	95% CI	*p* value	inter-group *p* value
Male							
No (142)	6.3 ± 1.9	5.4 ± 1.2	-0.9 ± 1.8	5.97	0.59–1.18	< 0.001	0.96
Yes (225)	6.4 ± 1.9	5.5 ± 1.3	-0.9 ± 1.6	8.00	0.66–1.09	< 0.001	
DM							
No (245)	6.3 ± 1.9	5.4 ± 1.2	-0.9 ± 1.7	8.19	0.79–1.12	< 0.001	0.67
Yes (122)	6.4 ± 1.9	5.6 ± 1.3	-0.8 ± 1.6	5.66	0.54–1.11	< 0.001	
CKD							
No (237)	6.0 ± 1.7	5.3 ± 1.2	-0.7 ± 1.5	7.32	0.51–0.89	< 0.001	0.012
Yes (130)	7.0 ± 2.0	5.8 ± 1.3	-1.2 ± 2.0	6.90	0.86–1.55	< 0.001	
Hyperuricemia at baseline							
No (207)	5.0 ± 1.0	5.0 ± 1.1	-0.0 ± 1.1	0.13	-0.14-0.16	0.90	< 0.001
Yes (160)	8.0 ± 1.4	6.0 ± 0.1	-2.0 ± 1.6	15.41	1.74–2.26	< 0.001	

*Abbreviations*: *SUA* serum uric acid, *SGLT-2i* Sodium glucose cotransporter-2 inhibition, *DM* diabetes mellitus, *CKD* chronic kidney disease (estimated glomerular filtration rate < 60 ml/min/1.73 m^2^)

## Disscusion

Our single-center retrospective study demonstrated that SGLT-2i use (primarily dapagliflozin) was associated with a significant reduction in SUA levels in patients with HFpEF, irrespective of comorbid DM or CKD. This benefit was observed without an increased risk of gout attacks or renal function deterioration and was more pronounced in patients with baseline hyperuricemia.

### SUA and HFpEF

Not exactly the same cause of traditional HFrEF, HFpEF may be related to myocardial abnormalities, myocardial stiffness, limited cardiac reserve, inflammation, pulmonary hypertension, and renal insufficiency [4]. Multiple previous studies have established associations between elevated SUA and cardiovascular disease, including AF [21], myocardial infarction [22], and coronary atherosclerotic disease (CAD) [23]. All of these are important risk factors for HFpEF, which could cause metabolic disorders, oxidative stress, and then accelerate the development of HFpEF [1, 4]. In Palazzuoli et al. ’s research [19], hyperuricemia is more common in HFpEF patients (57% in HFpEF vs. 43% in HFrEF, *p* = 0.01). Except the high incidence, hyperuricemia is also one of the risk factors for HF readmission and all-cause mortality for those patients [7, 19]. In our study, 734 patients with HFpEF were selected, among them about 82.2% of patients combined with two or more chronic diseases and 44.8% could be diagnosed with hyperuricemia, which was similar to the findings of Palazzuoli et al. [19].

Besides, patients with HFpEF usually performed as chest tightness, shortness of breath and increased volume load, which need diuretics to reduce volume load and improve the symptoms of HF. Diuretics, especially loop diuretics, can promote the reabsorption of uric acid [9], thus causing the increase of SUA and inducing acute gout attacks, which in turn could result in the difficulty in diuretic use and increase the risk of HF readmission [24]. To exclude the effect of diuretic on SUA, we focused on the dose of diuretic use during follow-up, and find no difference between groups.

### Urate-lowering therapy

Masami et al. [25] found that for HFpEF patients, the all-cause mortality increased significantly in the severe hyperuricemia group (SUA ≥ 8.3 mg/dL) compared to those with mild hyperuricemia (HR 1.73, *P* = 0.004), and ULT could significantly reduce it (HR 1.71, *P* = 0.041). The 2020 ACR gout guideline [26] recommends a target SUA level below 6 mg/dL, and for patients with high cardiovascular risk, SUA should be strictly controlled below 5 mg/dL. Notably, the URic acid Right for heArt Health (URRAH) study, a large retrospective analysis, provided prognostic SUA cut-off values specifically related to cardiovascular outcome events: >5.34 mg/dl for all HF and > 4.89 mg/dl for fatal HF [27].

Traditional ULT mainly include xanthine oxidase inhibitors (XOI) and uricosuric agents. The former is the first-line recommendation for guideline [26], such as allopurinol and febuxostat. However, there are some potential safety concerns with these drugs. Allopurinol may cause hypersensitivity reactions, especially in some Asians, which is considered to be associated with the HLA-B*5801 gene [26]. Meanwhile, no cardiovascular benefit was observed in prospective intervention study of allopurinol in hyperuricemia patients combined with HF [28]. Febuxostat is a non-purine XOI with better uric acid-lowering effect [29]. The EULAR study [29] showed that febuxostat (80-120 mg daily) reduced about 80% gout patients to SUA less than 6.0 mg/dl. However, Becker MA et al. [30]. showed that compared with allopurinol, febuxostat may increase the mortality in cardiovascular patients. It still requires further large-scale clinical studies to confirm the safety.

Therefore, there is still a large demand for clinical ULT and exploring cardiovascular drugs with concomitant urate-lowering effects is of clinical interest. Losartan, ARNI, and atorvastatin have been shown to reduce SUA [31–33]. Conversely, diuretics such as hydrochlorothiazide and spironolactone may elevate SUA by interfering with renal excretion [34]. Among them, SGLT-2i has shown promise in lowering SUA and improving cardiovascular outcomes in HF patients [16, 35, 36], positioning them as a novel therapeutic option worthy of further investigation in HFpEF populations with hyperuricemia.

### SGLT-2i lower SUA

As a novel hypoglycemic agent, SGLT-2i changes not only type 2 diabetes but also the management of HF [14]and CKD [12], and has now been recommended by multiple clinical practice guidelines [11, 13, 14]. SGLT-2i could inhibit the activity of the proximal S1 segment of renal tubules, reduce glucose reabsorption by renal tubular epithelial cells, then effectively induce urinary glucose excretion [14]. In addition to the hypoglycemic effects, large randomized clinical trials [17, 18] have shown that SGLT-2i could also continuously reduce SUA levels for patients with diabetes. The mechanism has not been clearly elucidated. The currently recognized mechanism is mainly through the expression of the glucose transporter 9 (GLUT 9) in the renal tubules, then causing the increased urinary excretion of glucose and uric acid [13, 18].

As to HF patients, several recently published clinical trials [16, 35, 36] yield similar conclusion that SGLT-2i could significantly improve clinical outcomes and reduce SUA level. The EMPEROR-REDUCE trial [16] also reveals that among those with the highest SUA level at baseline (mean 9.38 ± 1.49 mg/dl), the most decreased in SUA was observed after SGLT-2i use, with a mean reduction of 1.75 mg/dl. A meta-analysis [37] including 62 randomized controlled trials showed that different SGLT-2i could all significantly reduce SUA, especially dapagliflozin, in a dose-dependent manner—a finding that resonates with our study, where dapagliflozin was the predominant agent.

Studies mentioned above on SUA reduction by SGLT-2i were mainly focused on patients with diabetes and HFrEF, and pay less attention to HFpEF patients. In fact, the probability of combined hyperuricemia is higher in HFpEF patients because of more risk factors. In our cohort study, a retrospective analysis of SUA level before and after SGLT-2i use (primarily dapagliflozin) was performed in HFpEF patients in real world. Compared with control group, the SUA reduction was greater in SGLT-2i group (-0.91 mg/dl v s-0.1 mg/dl, *p* < 0.001). For the SUA target level of below 6.0 mg/dl required by the EULAR study [29], the control rate of SGLT-2i treatment could reach to 72.2% in our analysis, which was higher than the rate of control group (51.2%, *p* < 0.001) and was close to the febuxostat therapy in the EULAR study, the latter drug was used at relatively higher doses of 80–120 mg/d. In SGLT-2i group, compared with those without hyperuricemia, more SUA reduction was observed in those with hyperuricemia at baseline (-2.00 ± 1.64 mg/dl vs-0.01 ± 1.11 mg/dl, *p* < 0.001), which was consistent with the findings of the EMPEROR-REDUCE study [16].

### SGLT-2i and gout

Increased SUA can induce acute gout attacks, making it difficult to use diuretics, which could then increase the risk of HF readmission [9, 26]. Meanwhile, anti-gout drugs, such as nonsteroidal anti-inflammatory drugs and steroid hormones, may cause additional drug side effects, like gastrointestinal bleeding and renal injury. Therefore, 2021 ESC guideline for the management of HF [38] recommends avoiding such treatment in HF patients. Conventional urate-lowering drugs, such as allopurinol and febuxostat, could increase paradoxical acute attacks of gout at the beginning of drug use [39], increasing the use of the aforementioned anti-gout drugs, which is a major barrier to current standard gout treatment.

SGLT-2i reduces hyperuricemia without increasing acute gout attacks, and could even decrease the risk. In CANVAS trial [40], canagliflozin reduces the risk of gout attacks by 47% in DM patients. The DAPA study, which is focused on HF patients with dapagliflozin use, reaches the similar conclusion [15]. The post-hoc analysis [16] of EMPEROR-Reduced trial showed a mean SUA reduction of 1.12 mg/dL in empagliflozin group compared to placebo group. Compared to allopurinol, though the reduction in SUA was relatively small, gout events decreased by 32% with empagliflozin [35]. In our cohort study on HFpEF patients, the incidence of gout events during 3 to 6-month follow-up in SGLT-2i and control groups were 4.4% and 6.3%, respectively. This numerical difference suggests a potential protective tendency against gout attacks associated with SGLT-2i treatment (largely driven by dapagliflozin use), albeit not statistically significant.

### Subgroup analysis

In this study, we performed subgroup analysis according to gender, DM and CKD. Consistent with the finding of EMPEROR-Reduced trial, the effect of SGLT-2i on reducing SUA was consistent in different subgroups, including those with severe renal dysfunction.

### Hyperuricemia and CKD

SUA is an independent risk factor for patients with renal impairment [41]. However, many patients fail to achieve the target SUA level in clinical practice. While traditional first-line urate-lowering agents like allopurinol effectively reduce SUA [42], they have not demonstrated clear benefits in improving renal outcomes [43]. In contrast, SGLT-2i are proven to offer definitive renal protection, significantly reducing cardiorenal composite endpoint events and delaying progression to end-stage renal disease [44, 45]. This mechanism is thought to be related to tubuloglomerular feedback reducing intraglomerular pressure. Notably, the urate-lowering effect of SGLT-2i constitutes a significant part of its multifaceted benefits. Research confirms that this effect is independent of renal function or diabetic status; even in patients with advanced CKD, SGLT-2i can still lower SUA by increasing the uric acid excretion rate [46]. Data from the DAPA-HF trial also support this: in the approximately 41% of heart failure patients with an eGFR < 60 mL/min/1.73 m², the SUA reduction was similar to that in patients with normal renal function (-0.78 vs. -0.88 mg/dL, *p* = 0.30) [15].

Our study further strengthens this evidence: among the included HFpEF patients, 31.5% had concurrent CKD stages 3–5. Subgroup analysis showed that SGLT-2i significantly reduced SUA levels across different CKD stages. Importantly, our study found that patients with an eGFR < 60 mL/min/1.73 m² experienced a significantly greater reduction in SUA than those with an eGFR ≥ 60 mL/min/1.73 m² (-1.20 ± 1.99 vs. -0.70 ± 1.47 mg/dL, interaction *p* = 0.012). This finding suggests that for HFpEF patients with renal impairment, SGLT-2i may provide enhanced urate-lowering benefits.

### Hyperuricemia and DM

SGLT-2i, a class of glucose-lowering medications, induce urinary excretion of both glucose and uric acid, partly mediated by the expression of GLUT9 in renal tubules [13, 18]. Consistent with this mechanism, Ohashi et al. observed a significant increase in the fractional excretion of uric acid (from 5.98% to 7.71%) and a corresponding decrease in SUA levels (from 6.13 to 5.20 mg/dL) following SGLT-2i treatment in patients with DM [47]. Interestingly, several major cardiovascular outcome trials have suggested a more pronounced SUA-lowering effect in non-diabetic populations. For instance, the EMPEROR-REDUCE trial reported a greater mean reduction in SUA in non-DM patients compared to DM patients (-1.25 vs. -0.99 mg/dL, interaction *p* < 0.001) [35], a finding corroborated by the DAPA-HF trial (-0.95 vs. -0.70 mg/dL, interaction *p* < 0.001) [36].

In our study, baseline SUA levels were comparable between patients with and without DM (6.4 ± 1.9 vs. 6.3 ± 1.9 mg/dL). Diverging from the trial data above, we found that the SUA-lowering effect of SGLT-2i was similar between these groups during follow-up (-0.8 ± 1.6 vs. -0.9 ± 1.7 mg/dL, *p* = 0.67). This key finding underscores that SGLT-2i is an effective urate-lowering agent in HFpEF patients irrespective of diabetic status.

These findings hold direct relevance for clinical practice. While initiating dedicated ULT is not imperative for all hyperuricemia patients, especially those without frequent gout flares, a distinct therapeutic opportunity exists in HFpEF–especially emerging prognostic data from the URRAH study, which proposes stricter, outcome-driven SUA targets for HF. This population frequently presents with overlapping conditions such as diabetes and chronic kidney disease. Here, SGLT-2i provide a dual and synergistic benefit: they address the core cardiometabolic and renal comorbidities while concurrently and effectively lowering serum uric acid. This integrated pharmacologic action can simplify treatment regimens by reducing pill burden, achieve meaningful urate reduction, and may ultimately modify disease progression.

## Conclusions and prospects

In patients with HFpEF, SGLT-2i (primarily dapagliflozin) effectively lowers SUA levels without increasing the risk of gout attacks or renal impairment. This urate-lowering effect is consistent across key patient subgroups, including those with or without CKD and DM. Given the frequent comorbidity of hyperuricemia in HFpEF and the emerging prognostic importance of stricter SUA control, SGLT-2i offers a valuable therapeutic strategy that addresses both cardiovascular risks and uric acid metabolism concurrently. Future prospective studies are warranted to investigate whether the SUA reduction achieved with SGLT-2i, particularly towards more stringent prognostic targets, translates into incremental improvements in long-term cardiovascular outcomes.

### Limitations

This study has several limitations. Firstly, the single-center, retrospective nature of the study may have introduced selection bias. Secondly, the relatively short follow-up duration (3–6 months) may restricts the assessment of infrequent outcomes such as gout flares. Thirdly, as dietary intake significantly influences SUA levels, the lack of dietary data means its effect could not be adjusted for, representing a potential source of unmeasured confounding. Finally, the limitations of this study lie in the uneven distribution of the drugs in the SGLT-2i group, with dapagliflozin accounting for the majority. Future research should include more balanced data on various SGLT-2i to verify and expand these conclusions.

## Data Availability

Data is available upon request to the corresponding author.
